# Exploring Virtual Reality for Body Image Assessment and Psychological Interventions in Individuals With Obesity: a Comprehensive Review

**DOI:** 10.1111/obr.70051

**Published:** 2025-12-09

**Authors:** Giulia Corno, Ángel Zamora, Stéphane Bouchard, Rosa Maria Baños, Aurélie Baillot, Johana Monthuy‐Blanc

**Affiliations:** ^1^ Department of Psychoeducation and Psychology Université du Québec en Outaouais Gatineau Québec Canada; ^2^ Centre intégré de santé et des services sociaux de l'Outaouais Gatineau Québec Canada; ^3^ Research Unit Loricorps Centre de Recherche de l'Institut Universitaire en Santé Mentale de Montréal Montréal Canada; ^4^ Polibienestar Research Institute University of Valencia Valencia Spain; ^5^ Department of Personality, Evaluation and Psychological Treatment, Faculty of Psychology University of Valencia Valencia Spain; ^6^ CIBERObn Physiopathology of Obesity and Nutrition Instituto de Salud Carlos III Madrid Spain; ^7^ École interdisciplinaire de la Santé Université du Québec en Outaouais, Campus de Gatineau Gatineau Canada; ^8^ Institut du savoir de l'hôpital Montfort recherche Ottawa Ontario Canada; ^9^ Département sciences de l'éducation Université du Québec à Trois‐Rivières Trois‐Rivières Québec Canada; ^10^ Département de Psychiatrie et d’addictologie Université de Montréal Montréal Québec Canada

**Keywords:** body image, body image disturbances, obesity, review, virtual reality

## Abstract

**Introduction:**

Individuals living with obesity often experience body image (BI) disturbances, which can negatively affect their quality of life and treatment outcomes. Virtual reality (VR) has emerged as a promising tool for enhancing psychological interventions, but no comprehensive review has specifically focused on VR‐based studies addressing BI disturbances in this population.

**Methods:**

This comprehensive review examined studies utilizing VR for the assessment and treatment of BI disturbances in individuals with obesity. Twelve studies met the inclusion criteria.

**Results:**

Studies were categorized into three groups: (i) VR in psychological interventions for individuals with obesity, (ii) VR interventions following metabolic and bariatric surgery, and (iii) VR‐based full‐body illusion experiments. The primary clinical application was experiential cognitive therapy, which demonstrated greater efficacy in reducing negative BI compared with standard cognitive behavioral therapy and other treatments. Studies involving post‐metabolic and bariatric surgery adults also supported VR's efficacy in reducing BI dissatisfaction, though long‐term benefits were inconsistent. Full‐body illusion experiments suggested that VR can help modify distorted body perceptions. However, most studies were conducted by the same research group, focused exclusively on women, and were limited to specific geographical regions, primarily Italy.

**Conclusion:**

While preliminary results suggest that VR is a promising tool for treating BI disturbances in individuals with obesity, the field remains under‐researched. Notably, no studies have explored VR's potential as an assessment tool in this population. Future studies should include more diverse populations, investigate long‐term outcomes, and explore potential barriers to clinical implementation.

## 1 Introduction

Obesity is a complex and multifactorial condition, shaped by a combination of psychological, genetic, and environmental factors [1]. Its prevalence continues to rise globally, with nearly 40% of adults in the United States living with obesity [2, 3]. In Canada, the prevalence of obesity among adults reached 22% in 2022, representing approximately 8.7 million individuals [4]. In Europe, a recent report from the European Commission (2024) states that the proportion of individuals with overweight or obesity is rising quickly across many European countries, with 50.6% of people aged 16 and older in Europe classified as overweight in 2022. Beyond its well‐documented physical health implications, obesity is strongly associated with body image (BI) disturbances [5, 6, 7], which can significantly impact psychological well‐being.

BI disturbances typically encompass two key components: perceptual and attitudinal [8, 9, 10]. The perceptual component involves a distorted perception of body size, while the attitudinal component involves negative emotions, beliefs, and behaviors directed towards one's body [9, 10]. The first studies on BI disturbances in individuals with obesity date back to the 1960s [6, 11], with body dissatisfaction, defined as a negative cognitive and affective evaluation of one's own physical appearance, emerging as a particularly prevalent concern, especially among women [2, 6, 9]. Beyond its psychological toll, body dissatisfaction has been linked to future weight gain and increased body mass index (BMI) over time [2, 12, 13].

The literature on perceptual disturbances in individuals with obesity is mixed; some studies report significant distortions in body size perception, either overestimating or underestimating their body size [14, 15, 16, 17, 18, 19, 20], while other research finds no significant misestimations [19, 21]. Those who misperceive their body size are often more dissatisfied with their appearance, preoccupied with body concerns, and tend to avoid social situations due to their appearance compared with individuals of healthy weight [17, 19, 22, 23]. Furthermore, these perceptual inaccuracies may lead to higher rates of treatment discontinuation [15, 19]. Among individuals seeking metabolic and bariatric surgery, body dissatisfaction is closely associated with negative psychological outcomes, such as binge eating, depression, and low self‐esteem [24, 25]. Notably, about 20% of these patients identify concerns about their appearance as the primary motivation for seeking metabolic and bariatric surgery [24, 25].

Given the psychological burden associated with BI disturbances, various psychological interventions have been developed to address persistent BI disturbances [26, 27, 28], with cognitive behavioral therapy (CBT) being the most prominent. In recent years, innovative approaches using virtual reality (VR) have emerged as promising tools for the assessment and complementary treatment for BI disturbances. These VR‐based interventions allow individuals to engage with immersive environments that can enhance traditional therapeutic outcomes, offering new avenues for intervention with encouraging preliminary results [29, 30, 31, 32]. By providing a secure and adaptable space, VR enables individuals to gradually confront distressing or triggering scenarios—such as mealtimes or exposure to their own bodies—in highly personalized settings, and at their own pace [30, 33, 34]. These environments can evoke genuine emotional responses, enabling clinicians to gain a deeper understanding of the patient's emotional challenges and collaborate on strategies to effectively manage these responses [34].

Moreover, VR allows users to embody synthetic virtual bodies, facilitating experiences such as full‐body illusions (also called “body swapping,” “body swap illusion,” or “VR‐body swapping”), which provide disconfirming multisensory feedback that helps users iteratively update their internal body models [34, 35, 36]. By embodying virtual bodies that either closely replicate or modify their physical appearance, individuals can explore new perspectives on their body size, shape, or appearance, fostering a shift in self‐perception [34, 37].

Over the past decades, VR has been widely used in psychological and neurocognitive interventions, with growing evidence supporting its efficacy [38, 39, 40]. The increasing affordability and user‐friendly nature of VR systems have further bolstered their effectiveness as therapeutic tools. Since the late 1990s, VR has also been employed in the treatment of obesity [33], with increasing scientific interest leading to innovative applications [33, 41, 42, 43, 44]. A recent review by [33] examined the use of VR in the treatment of individuals with overweight and obesity. However, this review considered studies that targeted individuals with obesity and overweight in general, without delving into the specific assessment and treatment of BI disturbances. To our knowledge, no reviews to date have conducted an in‐depth analysis and discussion specifically addressing VR‐based studies focused on the assessment and treatment of BI disturbances in individuals living with obesity.

The present study aims to bridge this gap by providing a comprehensive review of the existing literature on VR as an assessment and treatment tool for BI disturbances in individuals living with obesity. By highlighting current findings and identifying areas of further investigation, this study seeks to stimulate both debate and research on this topic, ultimately contributing to improving therapeutic strategies for this population.

## 2 Methods

A comprehensive search on PubMed, Web of Science, and PsycInfo was conducted. The search keywords included the following: "body image" OR "body representation" OR "body dissatisfaction" OR "body awareness" OR "body perception" OR "body evaluation" OR "body distortion" OR "body image disturbance" OR "body size estimation" OR "body satisfaction" OR "body appreciation" AND "virtual reality" OR "extended reality" OR "augmented reality" OR "VR" OR "virtual simulation" OR "virtual environment" AND "obesity" OR "obese". These keywords were translated into Spanish and Italian in order to search for articles published in these languages. However, the research did not produce any articles in Italian or Spanish.

The following inclusion criteria were adopted: (a) papers published up to November 2023 in English, Italian, or Spanish language; (b) studies involving individuals living with obesity; (c) use of VR for assessment and/or intervention on BI‐related variables; (d) primary or original research using qualitative, quantitative, or mixed methods, or protocols of interventions about which primary feasibility and/or efficacy data have been published. Theoretical papers and literature reviews were excluded. Two review authors independently assessed all the potential articles, and any ambiguity or disagreement was resolved through assessment by a third review author.

## 3 Results

**FIGURE 1:**
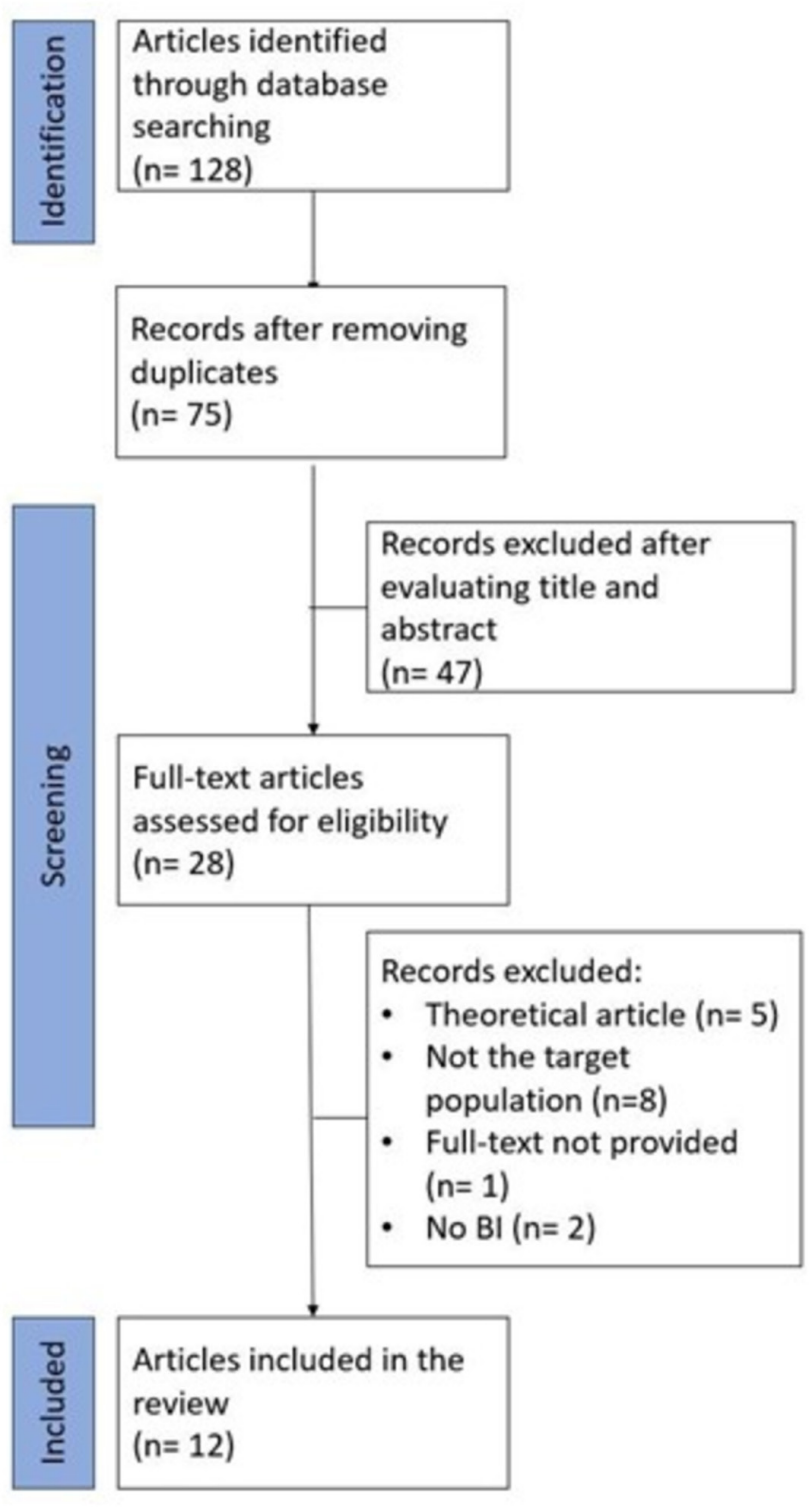
Flowchart depicting the identification and selection of the articles. BI, body image.

The initial search terms resulted in 128 articles. After deduplication, the title and abstract of 75 articles were screened, and 28 papers remained for further full‐text evaluation. Sixteen papers were excluded, and 12 articles met the specific criteria for inclusion in this comprehensive review (Figure 1).

Notably, three articles appear to have overlapping samples and reported the same corresponding outcomes [45, 46, 47] (Figure 1). Correspondence with the lead author confirmed that these three publications indeed refer to the same dataset. Consequently, they have been combined and treated as a single publication in this review. Tables 1, 2, and 3 outline details of the studies included, which were organized as follows: studies including VR as a tool in psychological treatment for individuals living with obesity (Table 1), studies including VR as a tool in psychological treatment for individuals who underwent metabolic and bariatric surgery (Table 2), and studies that used VR to perform a full body illusion experiment (also called VR‐body swap illusion) in individuals living with obesity (Table 3).

**TABLE 1:** Studies that involved VR in psychological interventions addressing BI disturbances in individuals living with obesity.

Article	Study design	Sample size, and characteristics	Treatment	Format	BI outcome measures	Other outcome measures	Main results end of treatment	Main result follow‐up
Cesa et al. [43] IT	RCT	*N* = 66 adult women with BED *n* = 27, ECT (BMI: *M* = 39.2, *SD* = 5.3) *n* = 20, CBT (BMI: *M* = 41.1, *SD* = 3.3) *n* = 19, Control (BMI: *M* = 40.8, *SD* = 6.3)	Inpatient setting Conditions: VR‐enhanced CBT (ECT) Standard CBT; Integrated multimodal medically managed impatient program (control)	Individual and group format 6 weeks ECT: 5 weekly group sessions and 10 biweekly individual VR sessions CBT: 5 weekly group sessions and 10 biweekly individual sessions over 5 weeks Control: low‐calorie diet, weekly nutritional groups, individual and group psychological support, and physical training	BSS; BIAQ; CDRS	Weight; BMI Number of binge eating episodes during the last month	Weight significantly decreased in all conditions, without any difference between them. Number of binge eating episodes decreased to zero in all the conditions. Body satisfaction increased in all the conditions, without difference between them. BI concerns significantly improved only in the ECT condition.	1‐year follow‐up Data available for 66.6% of participants (drop‐out rates similar for each condition). Statistically significant weight and BMI increases in the control group; no significant weight and BMI changes were found between post‐treatment and follow‐up in the ECT and CBT conditions. Across the three conditions, ECT participants showed statistically significant weight and BMI decreases at follow‐up. With respect to changes from baseline to follow‐up, ECT and CBT were better in improving or maintaining weight loss compared with the control condition. Number of binge eating episodes significantly increased in all participants from post‐treatment to follow‐up.
Manzoni et al. [44] IT	RCT	*N* = 158 adult women with morbid obesity BMI: *M* = 42.24, *SD* = 6.01	Inpatient setting Conditions: Standard behavioral program (SBP); SBT + CBT; SBT + VR‐enhanced CBT	Individual and group format 6 weeks SBP: medical, nutritional, physical, and psychological care, low‐calorie diet, physical exercise training CBT: 5 weekly group and 10 biweekly individual sessions VR‐enhanced CBT: 5 weekly group sessions and 10 biweekly VR sessions over 5 weeks	BSS; BIAQ; CDRS	Weight; DIET (description not available)	All participants reported a significant weight loss (% of weight loss: SBP 6.2%, CBT 7.4%, VR 6.25%). Eating behavior improved significantly for all participants; the VR group showed higher improvements. Body satisfaction improved significantly for all participants The VR group showed higher improvements.	One‐year follow‐up Data available for 113 patients the majority of patients who dropped‐out were in the SBP group, followed by CBT, and VR condition. When comparing pre‐treatment to follow‐up, only the VR group continued showing a statistically significant weight loss. When comparing post‐treatment to follow‐up, the SBP group showed a statistically significant increase in weight. The CBT showed a slight increase and the VR group no change. A significant difference was found between SBP and CBT and between SBP and VR group, but not between the CBT and VR condition. Patients in the VR condition had a greater probability of maintaining or further improve weight loss than patients in the SBP and CBT condition (VR: 48%, CBT: 29%, SBP: 11.5%).
Riva et al. [45, 46, 47] IT	Pre‐post intervention	*N* = 57 adult women *n* = 25, BED (BMI: *M* = 41.82, *SD* = 7.81) *n* = 18, BMI > 35 kg/m^2^ (BMI: *M* = 42.11, *SD* = 5.43) *n* = 14, EDNOS (BMI: *M* = 39.01; *SD* = 11.30)	Inpatient setting ECT	Individual and group format 6/8 weeks ECT: weekly psychological sessions, five VR sessions (VEBIM 2), low‐calorie diet, bi‐weekly psycho‐nutritional groups	BSS; BIAQ; FRS; CDRS	/	Body dissatisfaction toward the entire and body areas decreased significantly after the treatment for the BED group. In the group of women with a BMI > 35, body dissatisfaction toward the entire body decreased significantly, as well as dissatisfaction toward the limbs. Reduction in body dissatisfaction was less marked for the EDNOS group. Nevertheless, the treatment was able to induce a more realistic perception of the real body and an improved body satisfaction. Social activities related to BI improved significantly at post‐treatment for both groups. The use of disguising clothes decreased significantly at post‐treatment for both groups.	/
Riva et al. [48] IT	RCT	*N* = 28 adult women with a BMI > 35 kg/m^2^ and no history of purging within the six previous months *n* = n/a, experimental group (BMI: *M* = 43.50, *SD* = 5.97) *n* = n/a, control group (BMI: *M* = 44.44, *SD* = 10.07)	Inpatient setting Conditions: Experimental group; control group	Individual and group format 6.5 weeks Experimental: seven bi‐weekly VR‐based sessions (VREDIM), low‐calorie diet, and physical training (minimum two times a week) Control: low‐calorie diet, physical training (minimum two times a week), and psycho‐nutritional groups (three times a week)	BSS; BIAQ; FRS; CDRS	Weight; DIET; STAI; AI; WELSQ; URICA	Participants of the experimental group reported a reduced level of body dissatisfaction. The experimental group reported a significantly higher decrease in body dissatisfaction compared with the control group. Participants of the experimental group reported a reduced level of anxiety. The experimental group reported a significantly higher decrease in anxiety compared with the control group. Participants of the experimental group reported an increased self‐efficacy. Participants of the experimental group reported a decrease in overeating. Participants of the control group reported a significant change in the DIET Exercise score and in the AI ability and anxiety score. However, the reduction in the anxiety level was not confirmed by the STAI score. Participants of the experimental group experienced a mean weight reduction of 11.33 kg. Participants of the control group experienced a mean weight reduction of 7.58 kg.	/
Riva et al. [49] IT	RCT	*N* = 211 adult women with a BMI > 40 kg/m^2^ and one or more failures in following obesity treatment (BMI: *M* = 42.16, *SD* = 5.01) *n* = 56, ECT (BMI: *M* = n/a, *SD* = n/a) *n* = 52, CBT (BMI: *M* = n/a, *SD* = n/a) *n* = 50, NL (BMI: *M* = n/a, *SD* = n/a) *n* = 53, WL (BMI: *M* = n/a, *SD* = n/a)	Inpatient setting Conditions: ECT; CBT; nutritional group (NL); waiting list group (WL)	Individual and group format 6 weeks NL: 5 weekly nutritional groups, low‐calorie diet, physical training (minimum two times a week). CBT: five weekly nutritional groups, low‐calorie diet, physical training (minimum two times a week), five weekly group therapy sessions and 10 bi‐weekly individual therapy sessions. ECT: five weekly nutritional groups, low‐calorie diet, physical training (minimum two times a week), five weekly group therapy sessions, and 10 bi‐weekly VR sessions.	BSS; BIAQ; CDRS	Weight; DIET; STAI; WELSQ; EPI	A significant difference was found between the WL group and the three treatment groups in terms of weight changes. While no significant differences were found in the WL group, all the treatment groups were able to obtain significant weight reductions. Weight reduction was slightly higher in the CBT group compared with the NL and ECT group. Anxiety significantly decreased in all treatment groups, but not in the WL group. Eating behavior characteristics significantly improved in all treatment groups, but not in the WL group. Body satisfaction significantly improved in all treatment groups, but not in the WL group.	Six‐month follow‐up No participant was lost at follow‐up. Weight significantly decreased from pre‐intervention to follow‐up for the participants in all the three treatment conditions. No significant differences in the pre/follow‐up reduction were found between ECT and CBT group. However, the weight reduction for the ECT group was slightly higher. In the NT group, the follow‐up weight was higher than the weight at post‐treatment. In the ECT group, when compared with CBT: 25% more subjects achieved a 10% weight reduction, 11% more subjects were able to maintain or improve the weight reduction, and 8% less subjects reached a weight equal or higher than the initial one. The ECT group obtained significantly better improvements than both CBT and NT in the Neuroticism EPI scale. The ECT group obtained significantly better improvements than both CBT and NT in the head, torso, and global BISS. ECT obtained significantly better scores than the NT group in the Limbs BSS scale. The ECT group obtained significantly better improvements than both CBT and NT in the global BIAQ scores. ECT was significantly better than CBT in the Eating restraint BIAQ scale, and significantly better than NT in the Social activities BIAQ scale. The ECT group obtained significantly better scores than the NT group in the WELSQ total score.

Abbreviations: AI, assertion inventory; BED, binge eating disorder; BI, body image; BIAQ, Body Image Avoidance Questionnaire; BMI, body mass index; BSS, Body Satisfaction Scale; CBT, cognitive behavioral therapy; CDRS, Contour Drawing Rating Scale; DIET, Dieter's Inventory of Eating Temptations; ECT, experiential cognitive therapy; EDNOS, eating disorders not otherwise specified; EPI, Eysenck Personality Inventory; FRS, Figure Rating Scale; IT, Italy; NL, nutritional group; RCT, randomized controlled trial; SBP, Standard Behavioral Program; STAI, State–Trait Anxiety Inventory; URICA, University of Rhode Island Change Assessment Scale; VREDIM, Virtual Environment for Body Image Modification; VR, virtual reality; WELSQ, Weight Efficacy Life‐Style Questionnaire; WL, waiting list group.

**TABLE 2:** Studies that involved VR in psychological interventions addressing BI disturbances among individuals post‐metabolic and bariatric surgery.

Article	Study design	Sample size and characteristics	Treatment	Format	BI outcome measures	Other outcome measures	Main results end of treatment	Main result follow‐up
Cárdenas‐López et al. [50] MX	Case series	*N* = 3 Laparoscopic adjustable gastric banding adult patients *n* = 1 man (BMI: n/a) *n* = 2 women (BMI: n/a)	Unknown setting ECT	Individual and group format 6 weeks ECT: 15 sessions, five weekly group sessions and 10 biweekly individual VR sessions	BSQ	Weight; BDI; STAI; BULIT; TFEQ	Weight reduction between 15%–20% for all the participants. Improvement of the bulimic behaviors for all the participants. Anxiety decreased for all the participants, Improvement of body dissatisfaction for all the participants.	Three‐month follow‐up. Weight reduction between post‐treatment and follow‐up for all the participants. Improvement of bulimic behaviors for one participant; the other two participants showed a slight increase between post‐treatment and follow‐up. Anxiety decreased between post‐treatment and follow‐up for all the participants. Improvement of body dissatisfaction for two participants; one participant showed a slight increase between post‐treatment and follow‐up.
Cárdenas‐López et al. [51] MX	RCT	*N* = 24 metabolic and bariatric surgery adult patients Gender/sex: n/a *n* = n/a, WL group (BMI: *M* = 33.75, *SD* = 4.42) *n* = n/a, CBT group (BMI: *M* = 32.88, *SD* = 4.75) *n* = n/a, ECT group (BMI: *M* = 33.33, *SD* = 3.86)	Inpatient setting Conditions: CBT; ECT; WL	Individual and group format 12 weeks CBT: 15 sessions, weekly group and 10 biweekly individual sessions, over 6 weeks ECT: 12 sessions in 12 weeks	BSQ	BMI; BDI; IDARE; BULIT	No difference pre‐WL and post‐WL in terms of anxiety and depression; a significant decrease in anxiety and depression was found for the CBT and ECT condition. BMI significantly decreased in the ECT and CBT condition compared with the WL; no difference was found between ECT and CBT condition. BI improved in the CBT and ECT condition and worsened in the WL. Bulimia improved in the CBT and ECT condition and worsened in the WL.	Three‐month follow‐up N/A
Riva et al. [52] IT	Case study	One woman adult metabolic and bariatric surgery patient who experienced body dissatisfaction after the surgery (BMI after surgery: 33.38 kg/m^2^)	Unknown setting ECT	Individual and group format 6 weeks ECT: 5 weekly group therapy sessions, and 10 bi‐weekly individual VR sessions.	BSQ	Weight; BDI; STAI; BULIT; TFEQ	The patient reported a slight reduction of her weight. The patient reported an improvement in anxiety and depression. The patient reported an improvement in BI. The patient reported a reduction in the number of avoidance behaviors as well as an improvement in the number of adaptive behaviors related to eating. The patient reported an improvement in her physical and emotional well‐being (e.g., better skills in dealing with social eating situations, experienced more control over food), relevant behavioral changes in her personal and social daily life, and maintained with more facility the diet.	/

Abbreviations: BDI, Beck Depression Inventory; BI, body image; BMI, body mass index; BSQ, Body Shape Questionnaire; BULIT, bulimia test; CBT, cognitive behavioral therapy; ECT, experiential cognitive therapy; IDARE, State–Trait Anxiety Inventory; IT, Italy; MX, Mexico; RCT, randomized control trial; STAI, State–Trait Anxiety Inventory; TFEQ, Three Factors Eating Questionnaire; VR, virtual reality; WL, waiting list.

**TABLE 3:** Studies utilizing VR to implement full‐body illusions aimed at addressing BI disturbances in individuals living with obesity.

Article	Study design	Sample size and characteristics	Paradigm and conditions	BI outcome measures	Other outcome measures	Main results
Scarpina et al. [53] IT	Repeated measure	*N* = 30 adult women *n* = 15, BMI > 30 kg/m^2^ (BMI: *M* = 45, *SD* = 6.69) *n* = 15, BMI > 18.5 and < 25 kg/m^2^ (BMI: *M* = 22, *SD* = 1.66)	VR full body illusion (VR‐FBI): Exposure to a virtual body of a young slender woman from an egocentric point of view. Randomized order of the conditions. Conditions: Synchronous condition (experimental): visuo‐tactile stimulation on participants' abdomen, while a synchronous stimulation was delivered on the abdomen of the virtual female body; Asynchronous condition (control): visuo‐tactile stimulation on participants' abdomen, with a delay in the corresponding virtual touch.	Estimated and actual body measurements (height, shoulders, abdomen, hips) in terms of width and circumference; Percentage of misestimation.	EQ	All participants reported similar embodiment scores after the VR‐FBI, independently from the condition. All participants reported a stronger illusion in the synchronous condition compared with the asynchronous condition in terms of self‐location. Individuals with obesity had a significant overestimation of their height compared with the normal‐weight participants. This estimation did not change after the VR‐FBI for both groups. The synchronous illusion affected shoulder width estimation only of normal‐weight participants (increased underestimation). The VR‐FBI did not change the width estimation of the abdomen (which was underestimated in both groups) for both groups. Participants with obesity overestimated more the width of their hips compared with the normal‐weight participants, but the VR‐FBI did not change the width estimation of the hips for both groups. Normal‐weight participants overestimated more the circumference of their shoulders, and their abdomen compared with the participants with obesity, but the VR‐FBI did not change these estimations for both groups. After the synchronous illusion, all participants reported a reduction of the overestimation of the hips' circumference.
Serino et al. [54] IT	Case report	One adult woman BMI: 62.2 kg/m^2^	VR‐body swap illusion: Exposure to a virtual body of a young woman with a skinny belly in an egocentric point of view. Conditions: Synchronous condition: visuo‐tactile stimulation on participants' abdomen, while a synchronous stimulation was delivered on the skinny abdomen of the virtual female body; Asynchronous condition: visuo‐tactile stimulation on participants' abdomen, with a delay in the corresponding virtual touch.	BAT: Estimated and actual body measurements (height, shoulders, abdomen, hips) in terms of width and circumference; Average body perception indexes for width and circumference of the body size.	BES: Estimation of the distance (three types) between two tactile stimuli; Difference between the actual and estimated distance between the two tactile stimuli.	The participant reported sporadic unhealthy episodes of binge eating. The participant reported a tendency to devalue her body, strong body dissatisfaction, including concerns about body shape and lack of familiarity with her own body. The participant showed underestimation of the width of her body size. After the VR‐body swap illusion, the participant's body width estimations became more realistic. The participant presented an overestimation of her body circumference. After the VR‐body swap illusion, the ratio dropped to underestimation of the circumference of the body. The participant presented an overall overestimation of tactile distances. Mixed results were found regarding the ratios after the VR‐body swap illusion.

Abbreviations: BAT, body attitude test; BES, Body Eating Scale; BI, body image; EQ, embodiment questionnaire; IT, Italy.

### 3.1 Studies That Involved VR in Psychological Interventions Addressing BI Disturbances in Individuals Living With Obesity

Table 1 summarizes findings from five studies investigating the use of VR as part of a psychological treatment addressing BI disturbances among individuals living with obesity [43, 44, 45, 46, 47, 48, 49]. As mentioned before, three publications refer to the same dataset, so they are considered as one single study [45, 46, 47]. All studies were conducted in Italy by the same research group. All the five studies were conducted on samples of adult women in an inpatient setting. Among these, four were randomized controlled trials (RCT) [43, 44, 48, 49]. Out of all studies considered, two reported only pre‐treatment and post‐treatment results [45, 46, 47, 48] and three reported outcomes also at follow‐up [43, 44, 49], with follow‐up periods ranging from 6 months [49] to 1 year [43, 44]. Only one study included BI assessments at follow‐up [49]. All studies utilized the same self‐reported questionnaires (i.e., Body Satisfaction Scale, Body Image Avoidance Questionnaire) and figure rating scales. Additional variables examined included changes in weight [43, 44, 48, 49], BMI [43], number of binge eating episodes [43], anxiety [48, 49], eating behaviors [44, 48, 49], assertiveness [48], weight efficacy [48, 49], and changes in psychotherapy [48]. Notably, all studies appear to have employed the same psychological treatment or at least the same VR‐based module (i.e., VEBIM‐2/VREDIM) of this treatment: the experiential cognitive therapy (ECT). The ECT consists of five weekly group sessions focusing on eating, weight, and shape concerns and 10 biweekly individual sessions utilizing VR. During these sessions, therapists utilize 14 virtual environments depicting critical situations (e.g., supermarket, beach, restaurant) and BI comparison. These virtual environments are designed for practicing both emotional/eating/relational management, general decision‐making, and problem‐solving skills. By practicing these skills in VR, participants can develop effective strategies to manage and overcome triggering situations, which they can subsequently apply in real‐world scenarios [44]. Participants assigned to the ECT condition, or its VR module, reported improvements in BI measures [43, 44, 45, 46, 47, 48, 49] at post‐treatment and at follow‐up [49]. The comparison with other treatments and conditions led to the following main results: (a) ECT was more effective in reducing negative BI at post‐treatment than: standard CBT [43], a standard medically managed inpatient program [43], an intervention combining low‐calorie diet, physical exercise training and psycho‐nutritional groups [48], and a waiting list condition [49]; (b) ECT, standard behavioral program, and standard behavioral program combined with CBT showed comparable positive BI results at post‐treatment [44]; (c) participants assigned to the ECT reported significantly greater improvements in BI variables compared with those in standard CBT and nutritional intervention groups at 6‐months follow‐up [49].

In summary, the results of the identified studies indicate that ECT is a promising treatment for BI disturbances in individuals living with obesity. Participants generally reported improvements in BI outcomes both at post‐treatment and, in some studies, at follow‐up, with ECT often outperforming standard CBT, medically managed inpatient programs, or psycho‐nutritional interventions. The use of ecological virtual environments (e.g., supermarkets, restaurants, beaches) highlights the potential of VR to help patients rehearse strategies for real‐life triggering situations, offering practical applications for clinical care. Nevertheless, several methodological concerns limit the interpretability of these findings. Importantly, none of the studies included a stand‐alone BI treatment. In contrast, the extant studies employed a single comprehensive psychological treatment (which included the same VR‐based module) that targeted a wide range of outcomes, including eating behaviors and weight changes. This approach, while clinically common, limits the ability to isolate BI‐specific therapeutic effects and determine VR's precise contribution to observed improvements in BI outcomes. Secondly, the generalizability of the results from the included studies is constrained by the characteristics of the samples (only female participants), the sociocultural background (all studies were conducted by the same research group in the north of Italy), and the setting (only inpatient setting). These limitations raise important questions about replicability and clinical applicability across diverse populations and treatment contexts.

### 3.2 Studies That Involved VR in Post‐Metabolic and Bariatric Surgery Psychological Interventions Addressing BI Disturbances

Three articles investigated the integration of VR as part of a psychological treatment for adults who had undergone metabolic and bariatric surgery [50, 51, 52]. Two studies were conducted in Mexico and one in Italy. Among them, one study was a RCT involving adults after metabolic and bariatric surgery [51], another was a case series involving three adults (two women and one man) who underwent a laparoscopic adjustable gastric banding surgery [50], and the third was a single case study of an adult woman who underwent metabolic and bariatric surgery [52]. Information about the type of metabolic and bariatric surgery are not provided in two studies [51, 52]. The RCT was conducted in an inpatient setting [51], while no setting information was provided for the other two studies [50, 52]. Among the three papers, only the case series reported results pre‐treatment and post‐treatment and follow‐up [50]. The RCT mentioned a 3‐month follow‐up, but no results are reported [51]. Demographic data (e.g., gender/sex, age, BMI range) were not reported in the RCT. Additionally, there was no information on sample sizes per group or analyses exploring potential differences between participants assigned to the three conditions at pre‐treatment [51]. All studies utilized the Body Shape Questionnaire [55] as a self‐reported measure of BI. Additional variables assessed included BMI [51], weight loss [50, 52], depression and anxiety [50, 51, 52], and measures related to eating behaviors [50, 52] and bulimia [50, 51, 52]. All three studies examined the effects of the ECT. All three studies reported that VR integrated within ECT was effective in reducing BI dissatisfaction post‐treatment. Only the case series study reported BI changes at follow‐up, with mixed findings: Body dissatisfaction improved in two out of three participants [50]. In the RCT, ECT was compared against standard CBT and a waiting list condition [51]. The description of the CBT intervention was limited, as details about only 6 weeks on 12 weeks’ interventions are provided. Comparatively, both CBT and ECT were found effective in improving BI, while participants on the waiting list showed worsening BI over time [51].

In conclusion, ECT was once again the sole psychological treatment involving VR that was used among adults who had undergone metabolic and bariatric surgery. Despite the documented results of ECT's efficacy in enhancing BI, it is imperative to acknowledge the inherent methodological limitations that must be taken into account when interpreting these findings. First, it should be noted that a single RCT was conducted among adults who had undergone metabolic and bariatric surgery. Second, the generalizability of the results of the RCT is constrained by the paucity of information regarding important demographic characteristics of the sample, the type of surgery, randomization, and analyses to verify the possible presence of, and potentially control for, the effect of differences between participants in the three conditions before treatment. Moreover, it is not possible to argue about the long‐term effects of ECT, because information about the follow‐up assessment is not provided. Finally, these studies failed to address post‐metabolic and bariatric surgery‐specific BI concerns, particularly excess skin‐related dissatisfaction, which can represent a primary source of BI disturbance in this population [53].

### 3.3 Studies That Involved VR to Perform a Full Body Illusion to Address BI Disturbances in Individuals Living With Obesity

Two studies investigated the use of VR to implement a full body illusion task to address BI disturbances in individuals living with obesity [54, 56] (Table 3). Both studies were conducted in Italy. One study employed a repeated‐measure design [54], while the other was a case report [56]. The first [54] included 30 women, with 15 having a normal BMI, and 15 with obesity. The case report focused on an adult woman with severe obesity (i.e., obesity class III, corresponding to a BMI ≥ 40 kg/m^2^) deemed unsuitable for surgical intervention. Both studies utilized a similar VR‐based paradigm, despite differing terminologies [56]. Additionally, they could have employed the same virtual female body model, as evidenced by provided images showing substantial similarity between them. Specifically, participants in both studies underwent two types of visuo‐tactile stimulations (synchronous and asynchronous) on their abdomen while embodying a virtual slender female body in an egocentric, first‐person perspective. The virtual abdomen was stimulated (synchronously vs. asynchronously) with the participant's real abdomen. Scarpina et al. [54] specified the duration of each visuo‐tactile stimulation (90 s), while this detail was not provided in the study of Serino et al. [56]. Furthermore, Scarpina et al. [54] reported that the female virtual body was perceptively slimmer compared with participants of both the normal weight and obesity groups. Both studies measured estimated and actual dimensions of various body parts in terms of width and circumference, creating indices of misestimation. Both studies reported that participants living with obesity exhibited distortions in body part measurements. These distortions showed partial improvement following the VR task: a reduction of overestimation of hip circumference [53] and a more realistic estimation of the body width [56].

In summary, the results of these two studies indicate that VR could be used as a tool to specifically target distorted perceptions of body size in individuals living with obesity. These findings provide initial evidence that brief VR‐based body swapping interventions can partially modify perceptual distortions, suggesting a distinct therapeutic mechanism from the comprehensive cognitive‐behavioral approaches seen in ECT studies. However, the evidence base remains limited, and the observed improvements were limited to hip circumference reduction in one study and general body width estimation in the other, while width estimations for hips, abdomen, and shoulders remained unchanged in one study, raising questions about the clinical significance of such partial effects. The absence of follow‐up assessments prevents evaluation of whether these immediate perceptual changes translate into sustained improvements in BI satisfaction or real‐world functioning. Finally, the generalizability of the results is limited by the characteristics of the samples (only female participants), the sociocultural background (all studies were conducted in the north of Italy), and the use of the same virtual environment, suggesting these represent preliminary proof‐of‐concept findings rather than robust evidence for clinical implementation.

## 4 Discussion

Individuals living with obesity often experience BI disturbances, which are commonly addressed using psychological interventions, particularly those grounded in CBT [26, 27, 28]. In recent years, VR has emerged as a promising tool for enhancing the assessment and treatment of BI disturbances in this population. Yet, there has been no previous review that synthesizes the available knowledge regarding the use of VR for this purpose in individuals with obesity. Given this, the current study aims to fill this gap by providing the first comprehensive review of the existing literature on the use of VR in the assessment and treatment of BI disturbances among individuals with obesity.

The results reveal that the primary application of VR in treatments has been to enhance CBT. This has primarily led to the development of interventions such as ECT, including a VR‐based intervention, the VEBIM‐2 [45, 46, 47], and its enhanced version, VREDIM [48]. The ECT has shown to be more effective in reducing negative BI than standard CBT and other treatment modalities, such as medically managed inpatient programs and psycho‐nutritional interventions [43, 48, 49]. Furthermore, it has been demonstrated to be effective in enhancing positive BI, though not to a greater extent than other treatments [44]. Nevertheless, the long‐term outcomes of ECT showed to be more durable than those of alternative modalities that do not incorporate VR, such as standard CBT or nutritional interventions [49]. These findings suggest the potential of VR to enhance BI‐focused interventions for individuals with obesity.

Despite these promising results, several limitations must be considered when interpreting these findings. First, the number of studies is limited, with most being carried out by the same research group and some reporting results from overlapping samples. Second, the majority of studies have been conducted in Italy, focusing primarily on women with binge eating disorder or with severe obesity, while excluding men and other groups with varying demographic or weight characteristics or clinical contexts. These factors limit the generalizability of the findings to broader populations and different socio‐cultural contexts. Furthermore, the fact that ECT is the only intervention examined in multiple studies highlights the lack of attention this area of research has received in the scientific literature.

Among the studies reviewed, three studies specifically focused on adults who had undergone metabolic and bariatric surgery. In this subpopulation, ECT was the sole therapeutic approach employed, demonstrating effectiveness in reducing BI dissatisfaction post‐treatment [50]. However, it did not prove to be more effective than CBT [51], nor were consistent long‐term benefits observed [50]. As a result, while the findings suggest that ECT may be a promising therapeutic option, there is no clear data supporting its superiority over CBT. Furthermore, the studies did not account for the impact of significant weight loss and the resulting excess skin, which can contribute to BI dissatisfaction [57]. Finally, the presence of only one RCT, which also lacked sociodemographic information about the sample, further limits the conclusions regarding the efficacy of ECT [51].

As mentioned above, no study utilized a stand‐alone VR‐based treatment for BI. A stand‐alone BI treatment can be defined “as one where the BI image intervention was not combined with another extensive psychological therapy” [58]. The incorporation of BI‐specific interventions within comprehensive treatment programs is a common practice within this field. However, it has been highlighted how this practice can complicate the assessment of the effectiveness of specific BI interventions [28, 59]. This point is of particular importance in the context of studies conducted among individuals living with obesity. Given the dearth of literature on the subject, it is imperative to conduct studies that focus specifically on stand‐alone BI interventions among individuals with obesity. Such studies will facilitate the identification of interventions that are more suitable to the specific needs and characteristics of this population. Jerry and Berarbi [58] identified a list of 48 change techniques used in stand‐alone interventions to improve BI. The integration of VR technology in these change techniques holds considerable potential. For instance, it could be possible to recreate the virtual body of the individual and utilize it in an exercise designed to modify negative body language, address attentional bias towards weight‐related body parts, restructure thoughts and cognitions concerning the body, and engage in BI exposure exercises or social comparison exercises with other virtual bodies. Other VR‐based BI‐specific interventions for individuals with obesity could center on the identification and development of novel responses to weight‐stigma situations. It could be possible to argue: Why use VR as a tool to enhance BI interventions? There are numerous reasons. Primarily, VR allows for the generation of personalized scenarios and stimuli that can be customized to target individual triggers or disturbances [60]. Secondly, VR provides the opportunity to simulate, within a safe, confidential, and controlled setting, situations that may be difficult to recreate in real life and are often dependent on the individual's imagination [60, 61]. For instance, it could be possible to perform VR‐based exposure, also called in virtuo exposure, in a weight‐stigma situation, and work on emotional, cognitive, and behavioral responses, in an environment totally controlled by the researcher/therapist, ensuring also respect for the individual's pace. In this regard, in virtuo exposure is generally perceived as safer than in vivo exposure and can be more emotionally engaging than imagery‐based exposure [62].

Two studies explored the use of VR for full‐body illusion tasks to address BI disturbances [54, 56]. Both studies demonstrated the efficacy of a VR‐based paradigm in assessing and modifying distortions in body part perceptions. Specifically, they identified an overestimation of various body parts, with partial improvements observed after the VR task. Most notably, there was a reduction in the overestimation of hip circumference and a more accurate estimation of body width. These findings suggest that VR‐based full‐body illusion tasks may offer a novel approach to addressing BI disturbances in individuals with obesity. However, it is important to note that these results are based on only two studies, both conducted with women in the same country and using the same virtual environment, which limits their generalizability.

Interestingly, no study to date has utilized VR as an assessment tool to evaluate BI disturbances in individuals living with obesity. This is a surprising finding, since VR has been used to enhance visual‐perceptual methods (e.g., the paper‐based Stunkard Figure Rating Scale by [63]) to assess mental representations of the body (e.g., the perceived or ideal body size and shape) and BI disturbances (i.e., body dissatisfaction that is calculated from the discrepancy between perceived and ideal body size and body distortion that corresponds to the discrepancy between real and perceived body size) [64, 65]. Indeed, VR allows users to recreate the mental representation of an individual's perceived and ideal body, and to experience—or embody—this 3D, highly realistic body in both an allocentric (i.e., third‐person point of view) and egocentric (i.e., first‐person point of view) perspective. VR offers two primary methods for BI assessment: VR body size modulation tasks and VR body continuum tasks. In VR body size modulation tasks, users adjust virtual body parts to reflect their perceived or ideal body size [10]. On the other hand, VR body continuum tasks are similar to traditional paper‐based figure rating scales, where users, immersed in a virtual environment, are presented with a series of virtual bodies and asked to rate or select the one that best represents their body or their ideal body [64, 65, 66, 67]. These VR‐based BI assessment methods have been used with community (e.g., [10, 65, 66, 68]) and eating disorder samples (e.g., [67, 69, 70]). Future studies should consider the use of these advanced methods to assess the presence of BI disturbances in individuals living with obesity.

The results of this review also highlight a decline in research interest in recent years, as the last RCT in this area was conducted in 2016. Furthermore, the VR‐based application used as part of ECT, VEBIM‐2, which was once freely available online via the Neuro VR 2 platform (www.neurovr.org), is no longer accessible. The evidence remains preliminary, particularly for populations who have undergone metabolic and bariatric surgery. However, the use of VR in the field of mental health has continued to evolve, with growing evidence supporting its feasibility and acceptability across various clinical populations. Strong evidence already exists for the effectiveness and efficacy of VR‐based cognitive‐behavioral interventions especially for treating anxiety disorders, psychosis, and trauma‐related conditions (for a recent review, please refer to Bell et al. [70]). Emerging research also suggests potential benefits for eating disorders, depression, and stress reduction [70]. However, as with the application of VR for addressing BI disturbances in individuals living with obesity, more high‐quality randomized controlled trials are needed to conclusively establish the efficacy of VR‐based treatments across diverse populations.

These factors, along with the previously mentioned limitations, underscore the need for future research in this field. Future studies should aim to broaden the scope of investigation by including diverse cultural contexts, examining the effects of VR‐based treatments for BI on male populations, and exploring the BI challenges related to excess skin post‐metabolic and bariatric surgery. Additionally, multicenter studies with larger, more heterogeneous samples will be essential in validating these findings and enhancing the understanding of VR's role in improving and understanding BI in this population. Finally, it would be interesting for future studies to analyze the adverse effects or barriers to implementing VR in the clinical context, such as cost and long‐term adherence, which are essential for determining the feasibility and acceptability of its application in clinical practice for patients and clinicians. A potential future resurgence of interest in VR‐based BI studies among individuals living with obesity is a possibility, based on some factors. Firstly, the price of VR equipment has decreased in recent years, rendering this technology more affordable. Moreover, there is a growing recognition of the importance of addressing psychosocial factors, including BI, in the context of obesity [71]. As the understanding of the complex nature of obesity deepens, it has become imperative that interventions should extend beyond a narrow focus on sole weight loss to encompass the psychological and social dimensions of this condition.

The main strength of this comprehensive review lies in its exclusive focus on the use of VR for the assessment and treatment of BI disturbances in individuals with obesity. To date, no other review has specifically examined VR applications for this population, making this work a unique contribution to the literature. Additionally, this comprehensive review revitalizes interest in a field that has seen little progress in recent years, despite early evidence of the potential benefits of VR‐enhanced interventions, such as ECT. However, the review is constrained by the limited number of available studies, particularly regarding metabolic and bariatric surgery, many of which were conducted by the same research group, potentially introducing bias and restricting the diversity of findings. The absence of follow‐up data in most studies also limits the ability to draw strong, long‐term conclusions. Furthermore, the search was conducted using keywords in English, Spanish, and Italian. Therefore, the conclusions of this review are limited to scientific articles published in these three languages.

## 5 Conclusion

Individuals living with obesity often experience BI disturbances. CBT is the most common approach to address these issues. In the last few years, VR has been widely used in psychological interventions as a complement to CBT. In the field of eating disorders, this technology has demonstrated efficacy in assessing and improving BI. However, its specific application in individuals living with obesity remains underexplored.

This study provides an overview of the current literature on the use of VR for the assessment and treatment of BI disturbances in individuals with obesity. It underscores the importance of continued research in this area, as the findings suggest that VR is a promising tool for specifically treating BI disturbances among individuals living with obesity.

## Author Contributions

Conceptualization (GC, AZ, SB); methodology (GC, AZ, SB); data collection (GC, AZ); data curation (GC, AZ); formal analysis (GC, AZ, SB); writing – original draft (GC, AZ); writing – review and editing (GC, AZ, SB, AB, RB, JMB).

## Conflicts of Interest

J.M.‐B. has received honoraria for presenting research; she also receives royalties from books. S.B. is the President of, and owns equity in, Cliniques et Développement In Virtuo, a spin‐off company from the university that distributes virtual environments designed for the treatment of mental disorders. The terms of this arrangement have been reviewed and approved by the Université du Québec en Outaouais in accordance with its conflict‐of‐interest policies. SB has received honoraria for presenting research and giving workshops, and he also receives royalties from books. The remaining authors declare that the research was conducted in the absence of any commercial or financial relationships that could be construed as a potential conflict of interest.

## Funding

This research was supported by postdoctoral grants from the Foundation of the University Institute of Mental Health of Montreal affiliated with the Centre de Recherche de l'Institut Universitaire en Santé Mentale de Montreal, the Université du Québec à Trois‐Rivières, and the CIHR Fellowship (Health Research Training A‐Post‐PhD), the research funding from Social Sciences and Humanities Research Council (430‐2024‐00013) and research funding from the Fondation Santé Gatineau (FSG PR‐590183) awarded to GC; a grant (FPU20/05798) funded by the Spanish Ministry of Science Innovation and Universities awarded to AZ; AB gratefully acknowledges her Salary Awards from the Fonds de recherche du Québec‐Santé (FRQ‐S) (Chercheur Boursier Junior 2); the settlement fund of the Foundation of the University Institute of Mental Health of Montreal affiliated with the Centre de Recherche de l'Institut Universitaire en Santé Mentale de Montreal (2021–2025) and the Foundations of RBC Royal Bank and Takeda Canada (2021–2024) awarded to JMB.

## Data Availability

Data sharing not applicable to this article as no datasets were generated or analysed during the current study.
